# Texas Medical College

**Published:** 1873-05

**Authors:** 


*Texas Medical College.*

We are pleased to learn that the prospects for organizing this institution are encouraging. That it will be made a thorough success in every respect we have reason to believe. It has our best wishes in its organization, and for a future of usefulness.

